# D-dimer, BNP/NT-pro-BNP, and creatinine are reliable decision-making biomarkers in life-sustaining therapies withholding and withdrawing during COVID-19 outbreak

**DOI:** 10.3389/fcvm.2022.935333

**Published:** 2022-09-06

**Authors:** David M. Smadja, Benjamin A. Fellous, Guillaume Bonnet, Caroline Hauw-Berlemont, Willy Sutter, Agathe Beauvais, Charles Fauvel, Aurélien Philippe, Orianne Weizman, Delphine Mika, Philippe Juvin, Victor Waldmann, Jean-Luc Diehl, Ariel Cohen, Richard Chocron

**Affiliations:** ^1^Université de Paris-Cité, Innovative Therapies in Haemostasis, INSERM, Paris, France; ^2^Hematology Department, AP-HP, Georges Pompidou European Hospital, Paris, France; ^3^Université de Paris, Paris Cardiovascular Research Centre (PARCC), INSERM, UMR-S970, Paris, France; ^4^Hôpital Cardiologique Haut-Lévêque, Centre Hospitalier Universitaire de Bordeaux, Unité Médico-Chirurgical de Valvulopathies et Cardiomyopathies, Pessac, France; ^5^Medical Intensive Care Department AP-HP, Georges Pompidou European Hospital, Paris, France; ^6^Vascular Surgery Department, AP-HP, Georges Pompidou European Hospital, Paris, France; ^7^Emergency Department, AP-HP, Georges Pompidou European Hospital, Paris, France; ^8^Rouen University Hospital, FHU REMOD-VHF, Rouen, France; ^9^Institut Lorrain du Cœur et des Vaisseaux, CHU de Nancy, Vandoeuvre les Nancy, France; ^10^Université Paris-Saclay, Inserm, UMR-S 1180, Chatenay-Malabry, France; ^11^Cardiology Department, AP-HP, Georges Pompidou European Hospital, Paris, France; ^12^Cardiology Department, AP-HP, Saint Antoine Hospital, Paris, France

**Keywords:** COVID-19, life-sustaining therapies, withholding or withdrawing, ethics, claeys-léonetti law, biomarkers, SARS-CoV-2, mortality

## Abstract

**Background:**

The decision for withholding and withdrawing of life-sustaining treatments (LSTs) in COVID-19 patients is currently based on a collegial and mainly clinical assessment. In the context of a global pandemic and overwhelmed health system, the question of LST decision support for COVID-19 patients using prognostic biomarkers arises.

**Methods:**

In a multicenter study in 24 French hospitals, 2878 COVID-19 patients hospitalized in medical departments from 26 February to 20 April 2020 were included. In a propensity-matched population, we compared the clinical, biological, and management characteristics and survival of patients with and without LST decision using Student's *t*-test, the chi-square test, and the Cox model, respectively.

**Results:**

An LST was decided for 591 COVID-19 patients (20.5%). These 591 patients with LST decision were secondarily matched (1:1) based on age, sex, body mass index, and cancer history with 591 COVID-19 patients with no LST decision. The patients with LST decision had significantly more cardiovascular diseases, such as high blood pressure (72.9 vs. 66.7%, *p* = 0.02), stroke (19.3 vs. 11.1%, *p* < 0.001), renal failure (30.4 vs. 17.4%, *p* < 0.001), and heart disease (22.5 vs. 14.9%, *p* < 0.001). Upon admission, LST patients were more severely attested by a qSOFA score ≥2 (66.5 vs. 58.8%, *p* = 0.03). Biologically, LST patients had significantly higher values of D-dimer, markers of heart failure (BNP and NT-pro-BNP), and renal damage (creatinine) (*p* < 0.001). Their evolutions were more often unfavorable (in-hospital mortality) than patients with no LST decision (41.5 vs. 10.3%, *p* < 0.001). By combining the three biomarkers (D-dimer, BNP and/or NT-proBNP, and creatinine), the proportion of LST increased significantly with the number of abnormally high biomarkers (24, 41.3, 48.3, and 60%, respectively, for none, one, two, and three high values of biomarkers, trend *p* < 0.01).

**Conclusion:**

The concomitant increase in D-dimer, BNP/NT-proBNP, and creatinine during the admission of a COVID-19 patient could represent a reliable and helpful tool for LST decision. Circulating biomarker might potentially provide additional information for LST decision in COVID-19.

## Introduction

In the context of the coronavirus disease 2019 (COVID-19) outbreak, we were faced with the saturation of the health system, especially in emergency departments (ED) and intensive care units (ICU) (1–4). Consequently, physicians' priority was to have enough beds to hospitalize every patient who needed to be, and one of the ethical dilemmas faced by physicians during this pandemic concerned the decision of withholding or withdrawing life-sustaining treatments (LST). Prior to the COVID-19 outbreak, the decision of LST concerned mainly patients with chronic underlying diseases or lack of autonomy before the onset of an acute disease. Limitation of life-sustaining therapies is common worldwide with regional variability (5, 6). Indeed, the European Court of Human Rights (ECHR; 46 member states) pursuant to Article 2 of the Convention—that is, “the right to life”—considers a margin of appreciation as to whether or not to permit the withdrawal of artificial life-sustaining treatment and the detailed arrangements governing such withdrawal (7). According to the ECHR, administering or withdrawing medical treatment takes into account the following elements: (i) the existence of domestic law and practice of a regulatory framework compatible with the requirements of Article 2; (ii) whether account had been taken of the patient's previously expressed wishes and those of the persons close to him, as well as the opinions of other medical personnel; and (iii) the possibility to approach courts in the event of doubts as to the best decision to take in the patient's interests (8). In the United Kingdom, since July 2018 (9), the Supreme Court considers that whether or not withdrawing artificial life-sustaining treatment can be made without court approval unless “*it transpires that the way forward is finely balanced, there is a difference of medical opinion, or a lack of agreement from persons with an interest in the patient's welfare, a court application can and should be made*.” Indeed, the UK law states that life-sustaining treatment can be withdrawn without court approval if (i) the five principles of the Mental Capacity Act 2005 are followed (the principles are presumption of capacity, support to make a decision, ability to make unwise decision, best interest, and less restrictive option); (ii) relevant guidance is observed; and (iii) the question of what is in the patient's best interests is agreed. Last but not least, within the UK legal framework, an advance decision is a decision made by someone to refuse a specific type of treatment at some time in future. It gives guidance to the medical team to confirm the withdrawal of life-sustaining treatments, such as ventilation, cardiopulmonary resuscitation, and antibiotics (10). In France, decision of LST is regulated by the Claeys–Léonetti Law of 2 February 2016 recommending a collegial deliberation which must be reported in the medical file (11). The decision to LST is based on the discussion between the patients and/or their relatives and physicians. This new amendment provided new rights and reinforced others for patients and duties for medical professionals. Indeed, its article 1 reads that “*An obligation for health professionals to implement all the means at their disposal so that everyone has the right to have a dignified end of life accompanied by the best possible alleviation of suffering*.” Furthermore, advance directives from patients in relation to their life-sustaining treatments became binding on the medical teams (Article 5). This law, in its new version, underlines the importance of information given to the patient, and also it states that failing to have an advance directive, the medical team should consult with the designated trusted third party or the relatives. While decision of LST has been largely described within ICU worldwide, limited data exist in the ED or medical ward (MW) setting (12, 13). At the beginning of the COVID-19 outbreak, the unknown evolution of the disease and the very limited therapeutic options made LST decisions much more complicated. Therefore, both legal and ethical questions might arise from this peculiar and unique situation. First, we could wonder whether the legal framework is properly shaped in the time of pandemic. Second, from a mere ethical viewpoint, one could put into question physicians' ability to keep a clear and fair mind while undertaking a decision as serious as LST while under pressure, understaffed, and underequipped.

More than just a respiratory disease, severe acute respiratory syndrome coronavirus 2 (SARS-CoV-2) is a systemic acquired vascular disease associated with high thrombosis prevalence and/or a possible multiorgan failure (14). Several circulating plasma biomarkers have been described as interesting markers of initial severity, and most of them were prognostic of mortality and/or independent predictors of increased oxygen requirements (14–16). Markers of coagulopathy, in particular D-dimer, should therefore be helpful markers to improve the management of COVID-19 patients during hospitalization (17). As hospitals around the world were faced with an unprecedented influx of COVID-19 patients, the standard of care must be adapted to the health system capacity, and LST decision in MW and/or ED gave rise to ethical tensions. Thus, there was a need for a pragmatic risk stratification tool to help management and target resource allocation.

Using data from a multicenter French cohort (*n* = 2,848), we aimed to determine whether the biological profile at admission in hospital for COVID-19 could be associated with future LST and how it could be part of the prognosis elements the physicians could use every day to evaluate the appropriateness of an intensive, life-sustaining treatment.

## Methods

### Study settings and population

From 26 February to 20 April 2020, consecutive patients with a diagnosis of SARS-CoV-2 infection and initially hospitalized in medical wards were included (none of the patients were directly admitted in ICU). Patients were older than 18 years and were included in a retrospective, multicenter (24 centers), observational cohort study, which was named the Critical COVID-19 France (CCF) study and initiated by the French Society of Cardiology. Following World Health Organization (WHO) criteria, SARS-CoV-2 infection was determined by a positive result from a real-time reverse transcriptase–polymerase chain reaction (RT-PCR) test of nasal or pharyngeal swabs or lower respiratory tract aspirates (confirmed case) or by typical imaging characteristics on chest computed tomography (CT) when laboratory testing was inconclusive. The CCF study was declared to and authorized by the French data protection committee (authorization no. 2207326v0) and conducted in accordance with the ethical standards established in the Declaration of Helsinki and its later amendments (NCT04344327) (18, 19).

### Data collection

All datas were collected by local investigators in an electronic case report form *via* REDCap^®^ software (Research Electronic Data Capture^©^, Vanderbilt University, USA) hosted by a secured server from the French Institute of Health and Medical Research at the Paris Cardiovascular Research Center. Patients' baseline information included demographic characteristics, coexisting medical conditions, cardiovascular comorbidities, and chronic medications. Clinical parameters and biological findings were recorded at admission. On the chest computerized tomography (CT) scan, the degree of pulmonary lesions with ground-glass opacities and areas of consolidation was categorized as low/moderate (<50% involvement) or severe (≥50% involvement). Data on pharmacological therapies, mode of respiratory support, complications, and final vital status were also gathered throughout hospitalization. The time from hospitalization to death was used as an outcome. Outcomes were assessed using electronic medical records.

### Statistical analysis

To address confounding and other sources of bias arising out the use of observational data, we estimated a propensity-matched analysis for the likelihood of LST. We estimated the propensity score by running a logistic regression model where the outcome variable is a binary variable indicating LST decision, including the following as covariates: age, sex, body mass index, and history of cancer. Then, a 1:1 match was performed using greedy matching techniques. All analyses were performed on matched populations. We used the standardized mean difference (SMD), which is the most commonly used statistic to examine the balance of covariate distribution between the two groups (LST or no LST). Continuous data was expressed as median (interquartile range, IQR) and categorical data as *n* (%). All analyses were two-sided, and a *p* < 0.05 was considered statistically significant. Statistical analysis was performed using R studio^®^ software, including R version 3.6.3 (R Development Core Team, 2019).

## Results

### Withholding or withdrawing life-sustaining treatments practice based on medical records during COVID-19 outbreak

**Figure 1:**
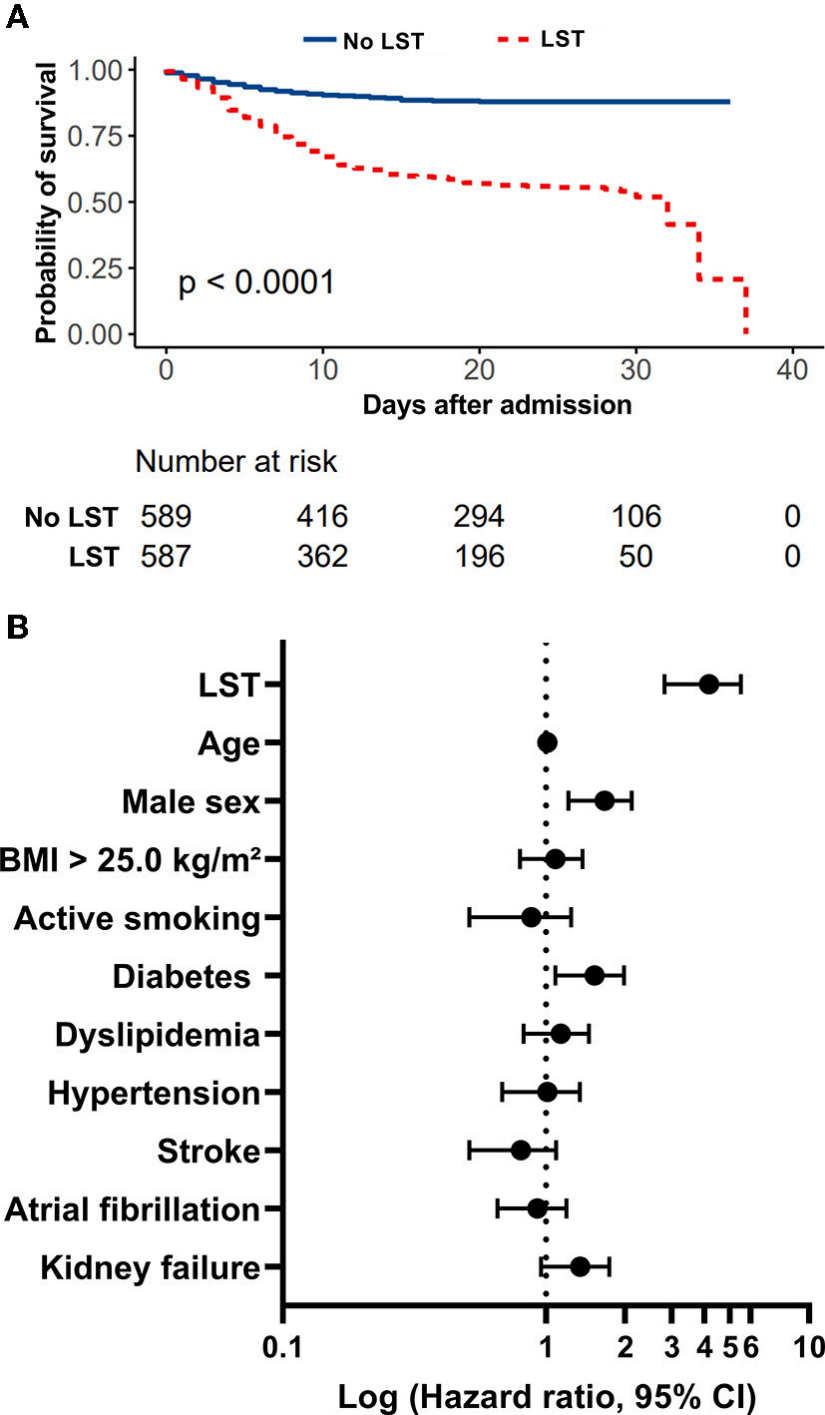
Evaluation of LST decision on in-hospital mortality in a retrospective study of COVID-19 patients admitted to medical wards during the first wave. **(A)** In-hospital mortality rate of patients with COVID-19 admitted to medical wards treated with or without LST decision. **(B)** LST is a strong predictor of in-hospital mortality in COVID-19 patients admitted to medical wards during the first wave. Forest plot summarizing results of Cox proportional-hazard model for in-hospital mortality adjusted on all covariates.

During the study period, 2,878 consecutive patients hospitalized for SARS-CoV-2 infection in medical wards were included, as previously described (1–3). Among the study population, an LST was decided for 591 COVID-19 patients (20.5%). These 591 COVID-19 patients with LST decision were secondarily matched (1:1) for age, sex, body mass index, and cancer history with 591 no-LST COVID-19 patients. As shown in Table 1, the study population had a mean age of 80.93 (±9.91) years, 568 patients (48.1%) were women, and the median body mass index was 26.18 (±5.01). LST patients had significantly more chronic cardiovascular diseases, such as high blood pressure (72.9 vs. 66.7%, *p* = 0.02), stroke (19.3 vs. 11.1%, *p* < 0.001), renal failure (30.4 vs. 17.4%, *p* < 0.001), and heart disease (22.5 vs. 14.9%, *p* < 0.001). Upon admission, LST patients were more severely attested by a qSOFA score ≥2 (66.5 vs. 58.8%, *p* = 0.03). In-hospital mortality occurred in 245 (41.5%) patients from the LST group and 61 (10.3 %) patients from the non-LST group, while time to death from admission in hospital was similar in both treatment groups (9.49 (±7.45) and 7.76 (±6.32) days, *p* = 0.06). We then realized a 40-day non-adjusted Kaplan–Meier curves for in-hospital mortality according to LST decision (Figure 1A), and we demonstrated statistically significant differences in in-hospital mortality (*p* < *0.001*). Finally, in Figure 1B, we demonstrated in a Cox regression model adjusted for all covariates (age, sex, BMI, active smoking) a significant difference of in-hospital mortality between LST and non-LST patients [adjusted hazard ratio: 14.03 (95% CI 2.90–5.58, *p* < 0.001)].

**Table 1:** Baseline characteristics of the population in a retrospective study for a matched population in LST patients with COVID-19 admitted in medical wards.

	**Entire population (*n* = 1,182)**	**Patients without LST decision (*n* = 591)**	**Patients with LST decision (*n* = 591)**	***p***
Age, mean (SD)	80.93 (9.91)	78.72 (8.69)	83.14 (10.56)	<0.001
**Gender**, ***n*** **(%)**				
Women	568 (48.1)	270 (45.7)	298 (50.4)	0.116
Men	614 (51.9)	321 (54.3)	293 (49.6)	
Body mass index, mean (SD)	26.18 (5.01)	26.39 (4.98)	25.98 (5.04)	0.162
Time from onset illness to hospitalization, mean (SD)	5.94 (4.87)	6.60 (4.92)	5.26 (4.72)	0.074
**Coexisting conditions**, ***n*** **(%)**				
History of cancer				
None	904 (76.5)	452 (76.5)	452 (76.5)	0.937
Active	141 (11.9)	67 (11.3)	70 (11.8)	
In remission	137 (11.6)	72 (12.2)	69 (11.7)	
High blood pressure	822 (69.8)	394 (66.7)	428 (72.9)	0.023
Diabetes mellitus	322 (27.5)	163 (27.7)	159 (27.2)	0.901
Dyslipidemia	423 (36.1)	207 (35.3)	216 (36.9)	0.596
Peripheral arterial disease	91 (7.8)	39 (6.7)	52 (8.9)	0.199
Ischemic stroke	178 (15.2)	65 (11.1)	113 (19.3)	<0.001
**Kidney failure**				
None	883 (76.1)	481 (82.6)	402 (69.6)	<0.001
Moderate (Cockcroft-Gault >30–60 mL/min/m^2^)	191 (16.5)	68 (11.7)	123 (21.3)	
Severe (Cockcroft-Gault <30 mL/min/m^2^)	63 (5.4)	26 (4.5)	37 (6.4)	
Critical (Hemodialysis)	23 (2.0)	7 (1.2)	16 (2.8)	
Kidney failure	277 (23.9)	101 (17.4)	176 (30.4)	<0.001
**Lung disease**				
None	985 (83.3)	498 (84.3)	487 (82.4)	0.493
COPD	97 (8.2)	44 (7.4)	53 (9.0)	
Asthma	45 (3.8)	25 (4.2)	20 (3.4)	
Chronic respiratory failure	55 (4.7)	24 (4.1)	31 (5.2)	
Current smoker	147 (12.8)	74 (12.8)	73 (12.8)	1.000
**Thromboembolism disease**				
None	1,038 (87.8)	532 (90.0)	506 (85.6)	0.039
Venous thrombosis	124 (10.5)	53 (9.0)	71 (12.0)	
Arterial thrombosis	20 (1.7)	6 (1.0)	14 (2.4)	
History of cardiomyopathy (any cause)	375 (32.1)	156 (26.7)	219 (37.6)	<0.001
History of atrial fibrillation	290 (24.8)	108 (18.4)	182 (31.2)	<0.001
**Characteristics at admission**, ***n*** **(%)**				
Extent of lung damage at CT scan in %				
<30%	3.34 (4.02)	197 (44.2)	210 (50.6)	0.133
30–50%	307 (26.1)	162 (36.3)	127 (30.6)	
>50%	188 (16.0)	87 (19.5)	78 (18.8)	
qsofa ≥2	531 (62.8)	237 (58.8)	294 (66.4)	0.028
**Medication history**, ***n (%)***				
ACE inhibitors	270 (22.8)	127 (21.5)	143 (24.2)	0.299
ARBs	242 (20.5)	139 (23.5)	103 (17.4)	0.012
Diuretic medication	350 (29.6)	151 (25.5)	199 (33.7)	0.003
Antiplatelet therapy	383 (32.4)	183 (31.0)	200 (33.8)	0.320
Statins	334 (28.3)	168 (28.4)	166 (28.1)	0.948
**Acute respiratory support**, ***n (%)***				
Optiflow	40 (3.4)	29 (4.9)	11 (1.9)	0.006
Non-invasive mechanical ventilation	27 (2.3)	11 (1.9)	16 (2.7)	0.436
Invasive Mechanical Ventilation	81 (6.9)	78 (13.2)	3 (0.5)	<0.001
**Outcomes**, ***n (%)***				
In-hospital mortality	306 (25.9)	61 (10.3)	245 (41.5)	<0.001
**In hospital mortality etiology**				
Acute respiratory failure	270 (89.4)	51 (83.6)	219 (90.9)	0.142
Acute cardiac failure	12 (4.0)	2 (3.3)	10 (4.1)	
Cardiac arrest	11 (3.6)	4 (6.6)	7 (2.9)	
Other	9 (3.0)	4 (6.6)	5 (2.1)	
Time to death, days, mean (SD)	8.10 (6.58)	9.49 (7.45)	7.76 (6.32)	0.066
**Final status**				
In hospital death	307 (26.1)	61 (10.4)	246 (41.8)	<0.001
Remain hospitalized in ICU	41 (3.5)	39 (6.6)	2 (0.3)	
Remain hospitalized in medical ward	188 (16.0)	86 (14.7)	102 (17.3)	
Discharged alive to home	473 (40.2)	327 (55.7)	146 (24.8)	
Discharged alive to rehabilitation center	167 (14.2)	74 (12.6)	93 (15.8)	

ACE, angiotensin-converting enzyme inhibitors; ARBs, angiotensin II receptor blockers; CT, computerized tomography; ICU, intensive care unit.

### Composite criteria of increased D-dimer, NT-pro-BNP/BNP, and creatinine highly predict decision of withholding or withdrawing life-sustaining treatments

**Figure 2:**
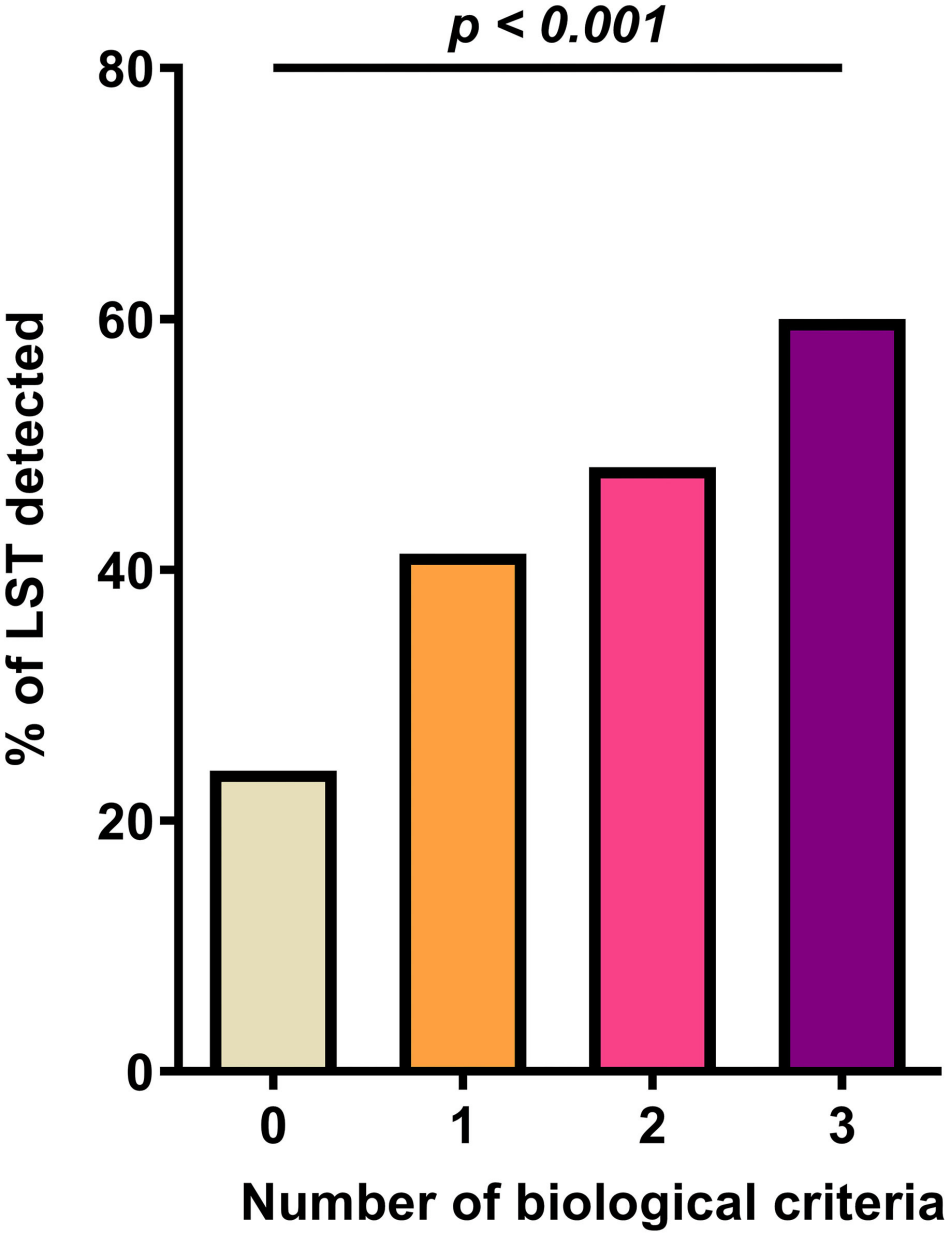
Composite biological criteria (D-dimer, NT-pro-BNP/BNP, and creatinine) help predict LST decision.

As shown in Table 2, LST patients had significantly higher values of D-dimer, markers of heart failure (BNP and NT-pro-BNP above and beyond clinical cutoff used in clinical practice), and renal damage (creatinine) on admission than the control group (*p* < 0.001). Their evolution was more often unfavorable (intra-hospital death) than the non-LST patients (41.5 vs. 10.3%, *p* < 0.001). Given the observation that D-dimer, BNP or NT-pro-BNP, and serum creatinine levels were increased in patients with LST decision, a ROC curve analysis was constructed using these biomarkers. While D-dimer, BNP or NT-pro-BNP, and serum creatinine level yielded weak AUC [56.3% (95% CI, 50.7–61.9) for D-dimer, 56.2% (95% CI, 52.3–60.1) for BNP or NT-proBNP, and 57.0% (95% CI, 53.7–60.3) for creatinine], a better AUC was observed for combined criteria with an AUC at 71.9% (95% CI, 65.5–78.4). These combined criteria with increased values for the three biomarkers had a sensitivity of 71.6% (95% CI, 61.8–81.4), a specificity of 66.3% (95% CI, 60.4–72.2), a positive predictive value (PPV) of 41.1% (95% CI, 33.0– 49.3), and a negative predictive value (NPV) of 87.6% (95% CI, 82.9–92.4%). Moreover, by combining the three biomarkers (D-dimer with cutoff of 1,128 ng/ml as previously described; creatinine above and beyond the median range), the proportion of LST increased significantly with the number of abnormally high biomarkers (24, 41.3, 48.2, and 60%, respectively, for none, one, two, and three high values of biomarkers; Figure 2, *p* < 0.001). Thus, using positive composite biological criterion should have helped LST decision in 60% of the case. Finally, we performed a logistic regression model to assess the association between LST decision and the composite biological criteria (D-dimer, NT-pro-BNP/BNP, and creatinine) adjusted on potential confounder [Table 3: adjusted for age, high blood pressure, ischemic stroke, kidney failure, thromboembolism disease, history of cardiomyopathy, history of atrial fibrillation (20), ARBs, diuretic medication]. After adjustment, the composite biological criteria remained significantly associated with LST decision, and this association increased with the number of criteria (adjusted OR of 2.75, 3.50, and 4.67 for 1, 2, and 3 criteria, respectively).

**Table 2:** Baseline biological profile of the population in a retrospective study for a matched population in LST patients with COVID-19 admitted in medical wards.

	**Patients without LST decision (*n* = 591)**	**Patients with LST decision (*n* = 591)**	***p***
White blood cells— × 109 per L	7.43 ± 6.07	8.14 ± 7.69	0.081
Hemoglobin—g/dL	12.84 ± 2.03	12.46 ± 2.14	0.002
Platelet count— × 109 per L	216.71 ± 99.81	209.27 ± 98.13	0.201
Plasma creatinine level—μmol/L	99.14 ± 80.66	120.13 ± 108.96	<0.001
MDRD—mL/min/m^2^	75.42 ± 28.37	65.90 ± 30.79	<0.001
Alanine aminotransferase—UI/L	36.94 ± 37.45	39.63 ± 68.19	0.422
bilirubin—umol/L	10.30 ± 5.86	12.69 ± 22.83	0.023
Gamma glutamyl transferase—IU/L	80.19 ± 106.95	89.43 ± 122.07	0.214
Alkaline Phosphatase—UI/L	92.95 ± 102.90	101.56 ± 78.86	0.141
Phosphate—mmol/L	0.96 ± 0.31	1.00 ± 0.34	0.204
calcium—mmol/L	2.21 ± 0.27	2.23 ± 0.21	0.154
Albumin—g/L	30.96 ± 6.17	30.20 ± 5.64	0.086
C-reactive protein—mg/L	89.88 ± 79.27	91.63 ± 76.33	0.704
Ferritin μg/L	879.77 ± 958.57	1056.25 ± 1666.33	0.262
Lactate dehydrogenase—UI/L	343.13 ± 151.97	355.30 ± 159.94	0.435
D-dimer—μg/L, *n* (%)			
<1,000 μg/L	86 (39.4)	49 (26.1)	0.004
1,000–2,000 μg/L	59 (27.1)	76 (40.4)	
>2,000 μg/L	73 (33.5)	63 (33.5)	
D-dimer ≥1,128 μg/L, *n* (%)	121 (55.5)	126 (67.0)	0.023
Fibrinogen—g/L	5.87 ± 1.65	5.71 ± 1.52	0.212
BNP—pg/mL	327.68 ± 739.75	592.33 ± 1117.90	0.017
BNP ≥500 pg/mL, *n* (%)	118 (72.0)	102 (85.7)	0.009
NT-proBNP—pg/mL	3421.19 ± 6433.58	5115.92 ± 10865.93	0.037
NT-proBNP ≥3,000 pg/mL, *n* (%)	168 (74.0)	262 (85.6)	0.001
BNP ≥500 and/or NT-proBNP≥3,000 pg/mL, *n* (%)	286 (73.1)	361 (85.5)	<0.001

Values are n (%) or mean ± SD.

**Table 3:** Analysis for LST decision in COVID-19 during the first wave of pandemic.

	**Adjusted OR (95%CI; *p*-value)**
**Composite biological criteria (D-dimer, NT-pro-BNP/BNP and creatinine)**	
0 Criteria	–
1 Criteria	2.75 (0.83–10.99, *p* = 0.117)
2 Criteria	3.50 (1.07–13.84, *p* = 0.050)
3 Criteria	4.67 (1.79–13.80, *p* = 0.003)
Age	1.02 (0.99–1.05, *p* = 0.126)
High blood pressure	1.14 (0.57–2.25, *p* = 0.707)
Ischemic stroke	2.23 (0.99–5.25, *p* = 0.059)
Kidney failure	1.42 (0.72–2.82, *p* = 0.306)
**Thromboembolism disease**	
None	–
Arterial thrombosis	2.45 (0.98–6.97, *p* = 0.068)
Venous thrombosis	1.41 (0.97–2.06, *p* = 0.074)
History of cardiomyopathy	1.75 (0.82–3.84, *p* = 0.154)
History of atrial fibrillation	0.89 (0.42–1.86, *p* = 0.754)
Qsofa ≥2	0.99 (0.56–1.76, *p* = 0.980)
ARBs	0.31 (0.16–0.61, *p* = 0.001)
Diuretic medication	1.08 (0.57–2.05, *p* = 0.824)

Logistic regression model to assess the association between LST decision and the composite biological criteria (D-dimer, NT-pro-BNP/BNP, and creatinine) adjusted on potential clinical confounder.

## Discussion

In this retrospective study, we demonstrated that a composite biological criterion, including D-dimer, NT-pro-BNP/BNP, and creatinine at admission in hospital, is linked to decision-making for LST in patients with COVID-19. Indeed, using a multicenter French study of patients hospitalized for COVID-19, we observed that LST patients evidenced by clinical criteria have higher in-hospital mortality than non-LST paired patients. Furthermore, in patients with positive composite biological criterion, LST was decided in 60%. Importantly, this is the first study exploring the usefulness of potential biomarkers in patients with COVID-19 by providing new objective criteria to help physicians decide on LST. Indeed, these criteria could potentially contribute to: (i) concretizing the medical decision by supporting clinical arguments with biological criterion and (ii) explaining objectives to the families while undertaking LST decisions. The COVID-19 pandemic has forced onto the medical community ethical questions combined with an unprecedented strain on the healthcare system, in particular in the emergency department and medical ward, to avoid transfer in intensive care units already overwhelmed. Decision of withholding or withdrawing life-sustaining treatments in emergency departments (ED) in France has been described mainly in patients older than 80 years with chronic underlying diseases, metastatic cancer, or previous functional limitations (12, 13, 21–24). Thus, our population is in line with decisions of withholding or withdrawing life-sustaining treatments usually carried out in emergency departments. Classically, decisions of LST in the ED and/or MW setting is mostly an extension at length within ICU worldwide. The ETHICUS 2 observational study confirmed considerable variations in end-of-life care practices across European intensive care units (6). This study reveals changes over the past decade in end-of-life practices: LST occurred more frequently, whereas deaths without LST are occurring less frequently and survival after LST is increased. Given the nature of the COVID-19 outbreak, the chaotic work environment in ED and MW could have been complicated and could have disturbed the availability to communicate and evaluate autonomy and/or advanced directives (25). Moreover, absence of family in hospitals due to health measures did not ease LST decision. This COVID-19 outbreak, particularly during the first wave between March and April 2020, induced an imbalance between medical, ethical, and public health concern. The clinical criteria used usually for LST in ED and/or MW are (i) evaluation of current failures in severity and numbers (hemodynamic, respiratory, neurologic); (ii) evaluation of chronic diseases (mainly tumor localization or dissemination, neurodegenerative disease); (iii) evaluation of loss of autonomy before acute disease; and (iv) advance directives if they exist and potentially associated with the additional opinion of the family when the directive is unclear or inexistent. Thus, LST decision is made on the basis of (i) probability of irreversibility of acute disease or impossibility to cure chronic disease in a short term; (ii) absence of improvement despite acute and invasive treatment; (iii) high probability of total loss of autonomy after current disease; (iv) major risk of total loss of autonomy after invasive treatment; and (v) advance directives coupled with families' observations and ethical approach. All these arguments, and in particular those concerning acute disease, were difficult to appreciate during the COVID-19 first wave since it was a new disease with several respiratory and cardiovascular complications, with uncertain evolution and with limited therapeutic options at this time. Thus, a non-subjective criterion like a biomarker level could have been a useful decision-making assistant to help appreciating severity and perspectives of patients with potential LST.

Biomarkers are used, with a bundle of arguments, to diagnose a disease and evaluate its severity or response to treatment strategies. Plasma biomarkers in COVID-19 have been largely evaluated to predict outcomes (mainly severity or in-hospital mortality) and could be classified into (i) biomarkers of immunological disorders and/or inflammation; (ii) biomarkers of hematological anomaly, and in particular hemostasis disorders; and (iii) biomarkers of organ injury, in particular cardiac and renal function markers. COVID-19 has been identified as a hypercoagulable state probably secondary to endothelial dysfunction following the inflammatory process and/or direct virus invasion (14). Our present study confirms that LST is associated with an increased level of D-dimer, NT-pro-BNP/BNP, and creatinine, respectively, related to coagulation activation, cardiac, and renal failure. A high level of these three pathways is the best predictor of LST in contrast to non-LST in our retrospective cohort of COVID-19 patients hospitalized in medical wards. We previously described in this multicenter cohort that a D-dimer concentration over 1,128 ng/mL is a relevant predictive factor for in-hospital mortality in patients hospitalized for COVID-19, regardless of the occurrence of venous thromboembolism during hospitalization (16). Here, we confirm relevance of coagulopathy in COVID-19 severity with increased D-dimer linked to the decision of LST. Cardiac dysfunction and renal dysfunction have also been largely described in COVID-19. Renal function impairment is highly present in patients with COVID-19 upon admission in hospital, and its severity is associated with higher mortality in this population (26). B-type natriuretic peptide (BNP) and N-terminal proBNP (NT-proBNP) are well-known markers of myocardial injury but are also increased in critical patients and in non-cardiac diseases, such as sepsis (27, 28). Thus, both cardiac and renal biomarkers are of interest in COVID-19 but are probably not enough to be specific to COVID-19. They are both associated with LST in contrast to non-LST in this cohort, and this global appreciation of organ function alteration could be a good marker even if they are not fully specific. Troponin I could be a better marker for cardiac injury since we and others have correlated increased troponin to severity and mortality in COVID-19 (15). However, troponin I was not strongly available in this cohort, and we will need to evaluate its interest in LST decision in another prospective cohorts in combination of other markers evaluated here. Our study assumes that coagulopathy, cardiac and renal failure could be jointly a marker of severity associated with LST decision. This biochemical point of view could be in line with a most severe cytokine storm. Indeed, inflammation in COVID-19 has been linked to these three symptoms (coagulopathy, cardiac and renal failure). While in our cohort, CRP is not discriminant in the LST decision strategy proposed here, different patterns of inflammatory cytokine could explain our results (29). Moreover, inflammation, coagulopathy, and secondary organ dysfunction could be all hallmarks of endothelial dysfunction (30). Endothelial disease has been proposed as a main trigger of COVID-19 severity (31) but also one of the main triggers of cardiovascular disease. Cardiovascular comorbidities, thrombosis, cancer, or neurological disorders can all be accompanied with endotheliopathy, and we need, in future, to explore endothelial biomarkers in LST decision not only in COVID-19 but also outside of COVID-19. Our study provides a new perspective by determining biomarkers as a potential decision-making helper of LST decision for COVID-19. The use of this combined biological criterion suggests there is a benefit to objective evaluation of coagulopathy and organ function to confirm LST decision that is often complex. It could ease the communication between patients, their family and/or relatives, and medical staff with objective and factual data. Moreover, several studies have pointed out ethical shortcomings in LST decision, in particular with different perceptions between physicians and nursing staff (32). Due to the fact that law in France requires consensus and a collegial decision, using biological criterion could avoid discrepancies among caregivers in the decision-making process and expedite processes of care in these unique circumstances of overwhelmed health system. The aim of this study is to reinforce the value of the medical decision and the relevance of the LST decision by bringing objective factors to the surface. Thus, this could contribute to nurturing a climate of trust between doctors, nurses, and families.

Our study has several limitations. Because of its retrospective design, it precludes determination of any causal relationship between LST decision and outcomes. Moreover, this study has included only patients who were initially hospitalized in medical wards and did not include patients who were directly admitted to the ICU. This selected population would not be representative of all patients with COVID-19, but more than that, our study cannot be applied to end-life patients with COVID-19 in ICU. Despite efforts to control confounders by using analytical strategies and matching method of propensity score, some potential biases may have been disregarded, such as clinical parameters involved in LST decision, in different centers since specific parameters have not been noted in the clinical registry of this study. Thus, by design, the study can only report associations.

All in all, our results highlight for the first time a beneficial and potential interest of biological criteria to help discriminate patients who could benefit from LST. When LST decision is proposed, we demonstrate that those patients are more severely sick with a worst biological evaluation of coagulopathy and organ function that could become a useful decision-making helper to decipher decisions and give good arguments to family to skip active therapies. Given the lack of consistency regarding LST decision, introduction of objectives biological parameters in patients with chronic disease or disabilities during COVID-19 infection could be largely beneficial for staff members involved in decision-making. Using biomarkers in LST needs to be tested in appropriate prospective randomized studies not only in COVID-19 but also outside of COVID-19.

## Data availability statement

The raw data supporting the conclusions of this article will be made available by the authors, without undue reservation.

## Ethics statement

The CCF study was declared to and authorized by the French Data Protection Committee (authorization no. 2207326v0) and conducted in accordance with the ethical standards established in the Declaration of Helsinki and its later amendments (NCT04344327). The patients/participants provided their written informed consent to participate in this study.

## Author contributions

DS and RC designed the present study. DS, BF, and RC wrote the manuscript. RC performed statistical analyses. AC and GB designed the trial. All the undersigning authors have substantially contributed to the article. All authors reviewed the article. All authors declare that the submitted work is original and has not been published before (neither in English nor in any other language) and that the work is not under consideration for publication elsewhere.

## Conflict of interest

RC, AC, and DS acknowledge the following without any relation with the current manuscript. RC received Consultant fees from Aspen. AC received research grant from RESICARD (research nurses) and consultant and lecture fees from Amgen, AstraZeneca, Bayer Pharma, Alliance BMS-Pfizer, Novartis, and Sanofi-Aventis. DS received consultant, lecture fees or travel awards from Aspen, Bayer, Carmat, Alliance BMSPfizer, Léo Pharma and Boehringer-Ingelheim. The remaining authors declare that the research was conducted in the absence of any commercial or financial relationships that could be construed as a potential conflict of interest.

## Publisher's note

All claims expressed in this article are solely those of the authors and do not necessarily represent those of their affiliated organizations, or those of the publisher, the editors and the reviewers. Any product that may be evaluated in this article, or claim that may be made by its manufacturer, is not guaranteed or endorsed by the publisher.
